# A Lethal Case of Cryptococcal Meningitis in an Apparently Immunocompetent Patient: A Case Report Highlighting Diagnostic Pitfalls and Therapeutic Challenges

**DOI:** 10.7759/cureus.98447

**Published:** 2025-12-04

**Authors:** Wye Hong Leong, Wee Fu Gan, Kok Tong Tan

**Affiliations:** 1 Infectious Disease Unit, Department of Medicine, Hospital Melaka, Melaka, MYS

**Keywords:** amphotericin b, antifungal therapy, cryptococcal meningitis, cryptococcus gattii, cryptococcus neoformans, hiv-negative, immunocompetent patients, lymphocytic pleocytosis

## Abstract

Cryptococcal meningitis (CM) is a relatively uncommon infection among immunocompetent individuals, with high rates of morbidity and mortality. We describe a 41-year-old seemingly immunocompetent gentleman with no significant past medical history who presented with fever, altered mental status, and headache for one week. The initial lumbar puncture (LP) exhibited lymphocytic pleocytosis with elevated cerebrospinal fluid (CSF) protein. CSF samples tested negative for common bacterial and viral pathogens; however, cryptococcal antigen was not tested. The patient was subsequently intubated for airway protection due to further deterioration in his mental status. Repeated CSF analysis tested positive for cryptococcal antigen with positive Indian ink microscopy. In spite of urgent surgical CSF diversion and combination antifungal therapy, the patient subsequently succumbed to his illness. This case highlights the great challenge in ensuring early and accurate diagnosis of CM, especially in immunocompetent patients who often present with indolent clinical manifestations. Greater emphasis should be placed on the importance of early diagnosis of CM and prompt initiation of effective treatment for better prognosis. This case underscores the critical need to include point-of-care cryptococcal antigen (CrAg) testing in the initial workup of all patients with unexplained lymphocytic pleocytosis, irrespective of their perceived immune status. Future research should be conducted to refine diagnostic approaches and treatment strategies for immunocompetent patients with CM.

## Introduction

Cryptococcal meningitis (CM) is an invasive, life-threatening fungal infection of the central nervous system that is most commonly caused by two main pathogens, *Cryptococcus neoformans* and *Cryptococcus gattii*. To date, CM remains one of the most common causes of opportunistic infections among people living with HIV (PLHIV), with approximately 223,000 newly diagnosed cases annually and at least 181,000 deaths globally [1]. *C. gattii* infection is more prevalent among immunocompetent patients with undefined risks as compared to *C. neoformans*, and the latter is said to be associated with more severe clinical manifestations, often resulting in hydrocephalus with delayed treatment response [2].

Traditionally, CM was described as predominantly affecting immunocompromised hosts, including PLHIV, individuals who are solid organ transplant recipients, patients who are on long-term chemotherapy, those with type two diabetes mellitus, and patients with rheumatological disorders such as rheumatoid arthritis (RA) and systemic lupus erythematosus (SLE) [2]. However, in recent years, the incidence of CM cases involving apparently immunocompetent individuals is increasingly being recognized, likely due to increased disease awareness and advancements in diagnostic capabilities.

CM is associated with high risks of mortality and morbidity with profound neurological sequelae due to its strong neurotropic properties and marked propensity to cause raised intracranial pressure, immune reconstitution inflammatory syndrome (IRIS), and disseminated fungal infection [3]. Previous studies have shown that even in immunocompetent individuals, the mortality rate of CM ranges from 9% to 15% in North America and Australia, whereas in low- and middle-income countries (LMICs) such as Sub-Saharan African countries, the mortality rate remains high, ranging from 22% to 70% [2,4]. Some literature has highlighted that HIV-negative patients with CM usually present with more atypical and indolent symptoms, which leads to significant diagnostic delays and subsequent poor clinical outcomes [5].

To date, Indian ink microscopy and cerebrospinal fluid (CSF) culture remain the gold standard for the diagnosis of CM. However, the definitive results may take up to one to two weeks, which may lead to unwanted diagnostic delays. Therefore, in recent years, many validation studies have advocated the identification of cryptococcal antigen (CrAg) in both serum and CSF, either by enzyme-linked immunosorbent assays (ELISA), lateral flow assay (LFA), or latex agglutination (LA) [6]. Point-of-care CrAg qualitative testing for CM has superseded the need for definitive CSF culture, and this has been shown to substantially improve the diagnostic yield and subsequent management of this life-threatening infection [6]. Other than that, neuroimaging techniques such as computed tomography (CT) and magnetic resonance imaging (MRI) of the brain are crucial prior to lumbar puncture (LP) to aid in making the diagnosis of CM. Some of the common radiological findings may include leptomeningeal enhancement, formation of cryptococcomas, dilated perivascular spaces, and hydrocephalus [7].

To our knowledge, there are limited case reports and reviews elucidating the comparison between immunocompromised and immunocompetent patients in terms of clinical characteristics and diagnostic accuracies of CM. As of now, there are no randomized controlled trials conducted for CM among non-HIV patients. In this paper, we report a case of an immunocompetent middle-aged gentleman who experienced a one-week history of acute confusional state, fever, and headache before being diagnosed with CM. We aim to emphasize the diagnostic difficulties encountered in an apparently immunocompetent patient with CM due to their atypical, indolent clinical presentations, and how these resulted in delayed diagnosis and subsequently, the patient’s mortality.

## Case presentation

A 41-year-old Malaysian Chinese gentleman with no prior medical illness presented to a private hospital in Malacca, Malaysia, in April 2024. He had a one-week history of fever, headache, photophobia, and altered mental status. He denied any intravenous drug use or any recent history of traveling. He worked as an operator in a noodle manufacturing factory.

On examination, he was initially confused when he arrived at the hospital, with occasional incoherent speech (day 0). The initial Glasgow Coma Scale (GCS) score on presentation was E4V4M6. However, he did not exhibit any signs of meningism, and there was no clinical evidence suggestive of opportunistic infection upon examination. Bilateral pupils were equal and responsive to light stimuli. His blood pressure on arrival was 131/90 mm Hg, pulse rate was 70 beats per minute, oxygen saturation on room air was 98% via pulse oximetry, and he was afebrile. The remainder of the neurological examination was unremarkable, and fundoscopic examination of both eyes was normal.

Initial blood investigations revealed leukocytosis, with a white cell count of 17.6 × 10⁹/L and elevated serum C-reactive protein (CRP) of 202 mg/L. Other biochemical parameters were unremarkable, with normal renal and liver function tests, as illustrated in Table 1. The patient also tested negative for hepatitis B, hepatitis C, and human immunodeficiency virus (HIV).

**Table 1:** Serial blood investigations of the patient throughout hospital admission.

Investigations	Day 0 (April 13, 2024)	Day 4 (April 17, 2024)	Day 10 (April 23, 2024)	Normal range
Full blood count				
Haemoglobin (mg/dL)	14.4	13.7	13.8	13.0–17.0
White blood cells (× 10^9^/L)	17.6	12.8	15.3	4.0–11.0
Neutrophil count (× 10^3^/ µL)	5.6	4.9	6.8	2.0–7.0
Lymphocytes count (× 10^3^/µL)	1.1	1.7	1.3	1.0–3.0
Platelets (× 10^9^/L)	396	260	81	150–400
Renal profile				
Serum urea (mmol/L)	3.4	6.3	19.5	2.8–8.1
Sodium (mmol/L)	130.0	131.0	157.0	135–145
Potassium (mmol/L)	4.2	4.5	3.7	3.5–5.2
Serum creatinine (µmol/L)	30.0	65.0	175.0	62–106
Liver function tests				
Alanine transaminase (U/L)	34	26	33	10–50
Aspartate transaminase (U/L)	24	29	56	10–50
Alkaline phosphatase (U/L)	58	83	96	40–130
Albumin (g/L)	40	32	29	35–52
Total serum bilirubin (µmol/L)	5.6	3.9	6.2	<24
C-reactive protein (mg/L)	202.0	283.5	258.3	˂5

Magnetic resonance imaging and angiography (MRI/MRA) of the brain (T2-weighted) demonstrated evidence of sulcal and leptomeningeal enhancement on post-IV gadolinium contrast enhancement, suggestive of meningitis. He was subsequently admitted for further evaluation and was empirically treated for meningitis with intravenous ceftriaxone 2 g every 12 hours and intravenous acyclovir 500 mg every eight hours.

A diagnostic LP was then performed in the ward, demonstrating a high opening pressure of 33 cm H₂O (normal: 6-18 cm H₂O). The macroscopic appearance of the CSF sample was clear and colorless. Further analysis revealed hypoglycorrhachia, with CSF glucose of 2.3 mmol/L, random blood glucose of 6.9 mmol/L, a CSF-to-serum glucose ratio of 0.33, and elevated CSF protein of 1.4 g/L, with lymphocytic pleocytosis. The initial CSF cell count was 335 cells/L (neutrophils 5%, lymphocytes 95%), and no organisms were seen on the CSF Gram stain. Indian ink staining was negative. CSF tested by the BioFire FilmArray Meningitis/Encephalitis (ME) panel was negative for 14 different bacterial and viral pathogens and for *Cryptococcus neoformans*/*Cryptococcus gattii* via nested polymerase chain reaction (PCR). Despite the high opening pressure and CSF profile suggestive of fungal meningitis or tuberculous meningitis (TBM), cryptococcal antigen testing was inadvertently omitted from the initial CSF workup, as the focus remained on bacterial and viral pathogens as well as TBM.

Two days later, he was transferred to the High Dependency Ward in Malacca General Hospital in view of financial constraints (day 2). A repeat LP was performed due to neurological deterioration, and CSF analysis revealed findings similar to the previous LP, as illustrated in Table 2.

**Table 2:** Cerebrospinal fluid analysis (CSF) throughout hospital admission. LFA: lateral flow assay, MTB/RIF: *Mycobacterium tuberculosis*/rifampicin resistance detection, VDRL: Venereal Disease Research Laboratory.

Investigations	Day 0 (April 13, 2024)	Day 4 (April 17, 2024)	Day 10 (April 23, 2024)
Opening pressure (cmH_2_O)	33	25	32
Macroscopic appearance	Clear	Clear	Clear
White blood cells (/µL)	335	250	134
Polymorphonuclear cells (%)	5	5	93
Lymphocytes (%)	95	95	7
Protein (g/L)	1.4	1.1	1.0
CSF glucose (mmol/L)	2.3	3.0	4.2
CSF/serum glucose ratio	0.33	0.5	0.7
Bacterial smear (Gram stain)	Negative	Negative	Negative
Bacterial culture	No growth	No growth	No growth
Fungal culture	Not done	No growth	No growth
Indian ink microscopy	Not done	Negative	Positive
Cryptococcus antigen (IMMY CrAg LFA)	Not done	Negative	Positive
*Mycobacterium tuberculosis* smear (Acid-fast stain)	Negative	Negative	Negative
*Mycobacterium tuberculosis* culture	Negative	Negative	Negative
GeneXpert MTB/RIF assay	Not done	Negative	Negative
VDRL test	Not done	Negative	Negative

**Figure 1:**
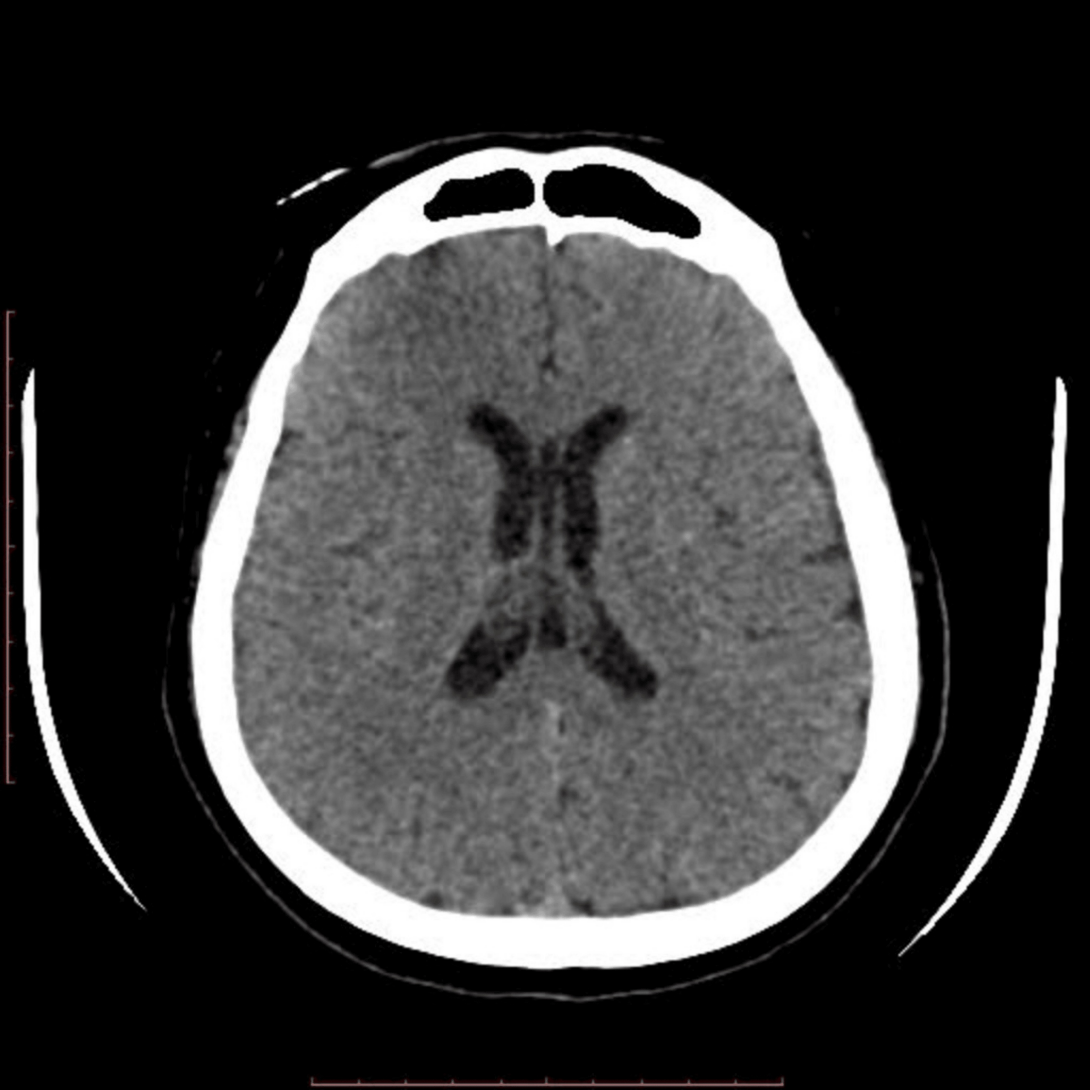
CT brain shows poor gray-white matter differentiation with sulcal effacement and mildly dilated ventricles, suggestive of cerebral edema and communicating hydrocephalus.

Seventy-two hours after admission, the patient’s condition further deteriorated, and he was intubated for airway protection, requiring vasopressor support (day 5). Repeat CT of the brain showed worsening cerebral edema and communicating hydrocephalus (Figure 1). The case was referred to the neurosurgical team for emergency surgical CSF diversion, and the patient subsequently underwent percutaneous placement of a lumbar drain, which was uneventful.

However, the CSF CrAg (IMMY LFA) obtained from the lumbar drain tested positive. Indian ink microscopy was also positive, demonstrating a few encapsulated yeasts. No cryptococcal antigenemia was detected, and both blood and CSF cultures did not yield any *Cryptococcus*. Urgent referral to the Infectious Disease (ID) team was made, and the diagnosis of CM was established after 10 days of hospital admission. The patient was promptly initiated on combination antifungal therapy with parenteral amphotericin B deoxycholate 60 mg every 24 hours (0.7 mg/kg/day) and oral flucytosine 2 g every six hours (25 mg/kg/dose) on day 10 of admission. Liposomal or lipid-based amphotericin B formulations were not available in our center, and thus the conventional deoxycholate formulation was used.

However, the clinical course was further complicated by hypernatremia and acute kidney injury. In spite of the antifungal treatment regimen and CSF diversion, he eventually succumbed to his illness due to multiorgan failure.

## Discussion

To date, the diagnostic dilemma in cases of CM remains a significant challenge, especially in patients with no clinical features suggestive of immunosuppression. This aligns with emerging evidence showing that unexpected neurological collapse in seemingly immunocompetent individuals can resemble other acute intracranial pathologies. Previous research has demonstrated how nontraumatic brain injury in sudden and unexpected deaths may clinically mimic infectious or inflammatory neurological conditions, underscoring the diagnostic challenges in this population [8]. Consequently, delays in diagnosis and treatment are usually associated with higher rates of morbidity and mortality even in immunocompetent patients. Many studies have emphasized the importance of maintaining a strong index of clinical suspicion for CM even in individuals who are immunocompetent, especially when high opening pressure is demonstrated in LP.

Notably, in this case, the patient presented with classic symptoms of CM, including fever, altered mentation, and photophobia. These clinical features were consistent with many case reports involving CM patients with no apparent immunodeficiency, as immunocompromised patients tend to present with gradual and subtle features, which may subsequently pose a great obstacle to early diagnosis [9]. Although immunocompetent patients with CM usually present with a subacute onset of prolonged headache, they may rarely exhibit atypical clinical manifestations such as stroke-like symptoms, unsteady gait, or visual or auditory symptoms [9].

While our patient had a negative HIV test and no overt history of immunosuppression, the designation of “apparent immunocompetence” must be interpreted with due caution. A definitive immunologic workup, including lymphocyte subset analysis and screening for autoantibodies against human granulocyte-macrophage colony-stimulating factor (GM-CSF), was not performed. Such underlying immunodeficiencies are increasingly recognized in patients presenting with cryptococcosis without the classical risk factors, and their presence may influence disease severity and outcome [10].

Therefore, it is reasonable to postulate that CrAg testing was not performed during the first hospitalization, likely because CM was not suspected as one of the possible differential diagnoses during the initial presentation, and there remained a high index of suspicion for TBM due to the insidious symptoms and CSF investigations. In Malaysia, where tuberculosis (TB) is endemic, TBM constitutes approximately 1-5% of all extrapulmonary TB cases, and its incidence is more common than CM [11].

Moreover, the CSF analysis exhibited lymphocytic pleocytosis with low CSF glucose and high CSF protein levels, which are classical CSF characteristics in patients with CM. However, differential diagnoses such as TBM, viral meningitis, or noninfectious lymphocytic meningitis still had to be considered. In our patient, the CSF workup was negative for TB or viral causes. Our case is consistent with one of the largest retrospective cohort studies conducted in Thailand by Rattagan et al. that compared clinical manifestations between HIV-positive and HIV-negative patients with CM [12]. This study showed that non-HIV-associated CM patients had significantly higher CSF leukocytes, lower CSF-to-blood glucose ratio, and higher CSF protein levels than those observed in immunocompromised patients, which may be due to a heightened immune response [12]. Elevated levels of CSF Th1 cytokines and downregulation of interleukin-6 inflammatory cytokines in immunocompetent individuals may be responsible for the low fungal burden and impaired fungal capsular production in CSF [13].

Due to lower fungal burden in immunocompetent patients, both latex agglutination tests and CSF cultures may be negative in cryptococcal infections, especially CM, which necessitates qualitative serum and CSF CrAg testing using LFA, as it has high sensitivity and specificity in symptomatic patients, even when tested among HIV-negative individuals, ranging from 95-99% and 96-99% respectively [14].

Moreover, the utilization of the BioFire ME panel may be limited in immunocompetent hosts, as this diagnostic assay has been found to have poor negative predictive value in patients with low disease burden [15]. Previous reports have concluded that a negative CSF cryptococcal PCR result is insufficient to exclude CM, especially in HIV-negative patients, and hence patients with a high index of suspicion for CM require additional CrAg testing [15]. In spite of this, the benefits of utilizing serum or blood CrAg in aiding diagnosis still do not supersede the importance of a diagnostic LP. Unfortunately, in this case, the initial omission of CrAg testing and the overemphasis on bacterial meningitis or TBM with negative CSF BioFire PCR results may have directly contributed to the delay in diagnosis.

Other than microbiological diagnosis, neuroimaging remained an essential adjunct for the diagnosis of CM and its potential complications. Several studies have shown that the neuroimaging features of CM in immunocompetent patients may be distinct as compared to those seen in immunocompromised individuals. In CM patients with no apparent immunodeficiency, gyriform leptomeningeal enhancement with basal cisternal predominance, dilatation of periventricular spaces, and formation of cryptococcomas were some of the commonly observed radiological features on contrast-enhanced MRI of the brain [7]. However, it is essential to note that there were no radiologically defining characteristics that consistently differentiated immunocompetent from immunocompromised patients with CM [7,16].

Management of CM is complex and requires a multifaceted, multidisciplinary approach, irrespective of a patient’s immune status. This includes early consultation with the infectious disease team for effective combination antifungal therapy, urgent referral to the neurosurgical team for surgical intervention of elevated intracranial pressure (ICP), and anticipation and management of IRIS.

The antifungal strategy for CM mainly comprises three phases: induction, consolidation, and maintenance. Recent WHO guidelines in 2022 recommended a single high-dose liposomal amphotericin B in combination with other standard medications (flucytosine or fluconazole) as part of induction therapy for PLHIV, which aids in rapid sterilization of CSF due to potent fungicidal activity. In the absence of specific clinical guidelines for HIV-negative patients, we adhered to the induction regimen recommended by both the Infectious Diseases Society of America (IDSA) and the Malaysia National Antimicrobial Guideline (2024) for CM, which consists of amphotericin B and flucytosine [17].

Complications such as raised ICP and hydrocephalus are common in both PLHIV and HIV-negative individuals with CM, and are associated with high risks of morbidity and mortality. Therapeutic options include serial therapeutic LPs and surgical CSF drainage, which can be performed via external ventricular drain or lumbar drain insertion. Multiple studies have shown that early initiation of effective fungicidal therapy and prompt surgical CSF diversion are associated with significant reductions in mortality in both immunocompetent and immunocompromised patients with CM [18].

Interestingly, several studies have shown that non-HIV CM patients have a greater propensity for hydrocephalus and higher rates of neurosurgical intervention, as seen in this case. However, the mechanisms underlying this phenomenon are poorly understood [10,18].

Conversely, there is conflicting evidence on clinical outcomes between immunocompetent and immunocompromised CM patients. However, most studies have demonstrated that non-HIV, nontransplant CM patients are associated with higher 90-day mortality and more unfavorable clinical outcomes, which may be attributed to delayed or initially incorrect diagnosis and hence delayed treatment initiation [19]. Some of the poor prognostic indicators in this group include altered mental state at presentation, age over 50 years, presence of raised intracranial pressure (CSF opening pressure >25 cm H₂O), and presence of cryptococcal antigenemia [20]. It is vital to remember that both immunocompetent and immunosuppressed patients are susceptible to CM, and prompt effective treatment should be initiated for better clinical outcomes.

## Conclusions

In summary, this case highlights the importance of maintaining a high index of suspicion for CM as a possible cause of meningitis even in immunocompetent patients. In recent years, there has been rising recognition that CM can also affect immunocompetent individuals, with high rates of mortality and morbidity due to inherent diagnostic delays and failures to administer timely treatment. Hence, early identification of symptoms and diagnosis with the appropriate combination antifungal therapy are paramount in improving the clinical outcomes of patients with CM. However, it is still crucial to maintain a broad differential diagnosis in all patients who present with clinical and biochemical features of meningoencephalitis.

More clinicians should be aware of the diagnostic limitations of the various microbiological methods used, as negative results do not conclusively exclude CM. Point-of-care CrAg testing provides a rapid qualitative result that can be performed on both serum and CSF samples. These diagnostic assays have high specificity and sensitivity in symptomatic patients and therefore should be performed in all patients with suspected CM. Future areas of research should focus on microbiological and radiological diagnostic modalities to help clinicians promptly recognize and manage CM in both immunocompetent and immunosuppressed populations.
